# The extracellular matrix of mycobacterial biofilms: could we shorten the treatment of mycobacterial infections?

**DOI:** 10.15698/mic2019.02.667

**Published:** 2019-01-18

**Authors:** Poushali Chakraborty, Ashwani Kumar

**Affiliations:** 1 Council of Scientific and Industrial Research, Institute of Microbial Technology, Chandigarh, India 160036.; 2 CSIR-Academy of Scientific & Innovative Research (AcSIR), Council of Scientific & Industrial Research, New Delhi-110001.

**Keywords:** extracellular polymeric substance, pellicle biofilm, macrocolony biofilm, thiol reductive stress, cellulose, extracellular matrix, biofilms, Mycobacterium, tuberculosis pathogenesis

## Abstract

A number of non-tuberculous mycobacterium species are opportunistic pathogens and ubiquitously form biofilms. These infections are often recalcitrant to treatment and require therapy with multiple drugs for long duration. The biofilm resident bacteria also display phenotypic drug tolerance and thus it has been hypothesized that the drug unresponsiveness *in vivo* could be due to formation of biofilms inside the host. We have discussed the biofilms of several pathogenic non-tuberculous mycobacterium (NTM) species in context to the *in vivo* pathologies. Besides pathogenic NTMs, *Mycobacterium smegmatis* is often used as a model organism for understanding mycobacterial physiology and has been studied extensively for understanding the mycobacterial biofilms. A number of components of the mycobacterial cell wall such as glycopeptidolipids, short chain mycolic acids, monomeromycolyl diacylglycerol, etc. have been shown to play an important role in formation of pellicle biofilms. It shall be noted that these components impart a hydrophobic character to the mycobacterial cell surface that facilitates cell to cell interaction. However, these components are not necessarily the constituents of the extracellular matrix of mycobacterial biofilms. In the end, we have described the biofilms of *Mycobacterium tuberculosis* (Mtb), the causative agent of tuberculosis. Three models of Mtb biofilm formation have been proposed to study the factors regulating biofilm formation, the physiology of the resident bacteria, and the nature of the biomaterial that holds these bacterial masses together. These models include pellicle biofilms formed at the liquid-air interface of cultures, leukocyte lysate-induced biofilms, and thiol reductive stressinduced biofilms. All the three models offer their own advantages in the study of Mtb biofilms. Interestingly, lipids (mainly keto-mycolic acids) are proposed to be the primary component of extracellular polymeric substance (EPS) in the pellicle biofilm, whereas the leukocyte lysate-induced and thiol reductive stress-induced biofilms possess polysaccharides as the primary component of EPS. Both models also contain extracellular DNA in the EPS. Interestingly, thiol reductive stressinduced Mtb biofilms are held together by cellulose and yet unidentified structural proteins. We believe that a better understanding of the EPS of Mtb biofilms and the physiology of the resident bacteria will facilitate the development of shorter regimen for TB treatment.

## INTRODUCTION

Bacteria are generally studied in the research laboratories as single cell suspensions called as planktonic cultures, however, in nature, bacteria primarily exist as a community encased in a self-produced extracellular matrix called as biofilms. There are many advantages of studying bacteria in the planktonic cultures such as development of a homogenous population of bacterial cells having similar transcriptomic, proteomic and metabolomic profile etc. But the bacterial growth in biofilms requires a varied but coordinated transcriptional, proteomic and metabolomic profile. The bacterial cells residing in biofilms exhibit quite different phenotypic properties compared with their planktonic counterparts [1]. Formation of bacterial biofilms requires cooperation, differentiation and division of labor, capturing and sharing of resources such as nutrients. Microbial biofilms ensure improved survival following exposure to antimicrobials and physicochemical stresses. Bacteria residing in biofilms are highly heterogeneous and understanding their physiology is challenging. Given the physiological heterogeneity, the biofilm resident bacteria depict phenotypic drug tolerance that is of relevance for a number of infections. Bacterial biofilms are associated with a number of infections such as endocarditis, cystic fibrosis, pneumonia, infectious kidney stones, inner ear infections and many hospital-acquired infections from catheters and ports [2-4]. Biofilm resident bacteria display 100-1000 folds higher minimal inhibitory concentration (MIC) as compared to planktonic bacteria making their treatment a challenging task [5]. It is believed that the extracellular polymeric substance (EPS) could act as a barrier for antibiotic penetration and thus may contribute to the drug tolerance observed in biofilms. The basic ultrastructure of the bacterial biofilms largely depends on the extracellular matrix produced by the cells within the biofilms. The matrix of biofilms is composed of different types of biopolymers known as EPS. In most of the bacterial biofilms, most of the dry mass is due to EPS, while bacteria contribute only to a small fraction of the total dry mass [6]. EPS provides mechanical stability to biofilms through physiochemical interactions that involve electrostatic forces, hydrogen bonds and van der Waals interactions [6, 7]. Although the composition of EPS varies significantly among different bacterial species, extracellular polysaccharides, proteins and lipids remain as the key components of EPS [8].

**Figure 1:**
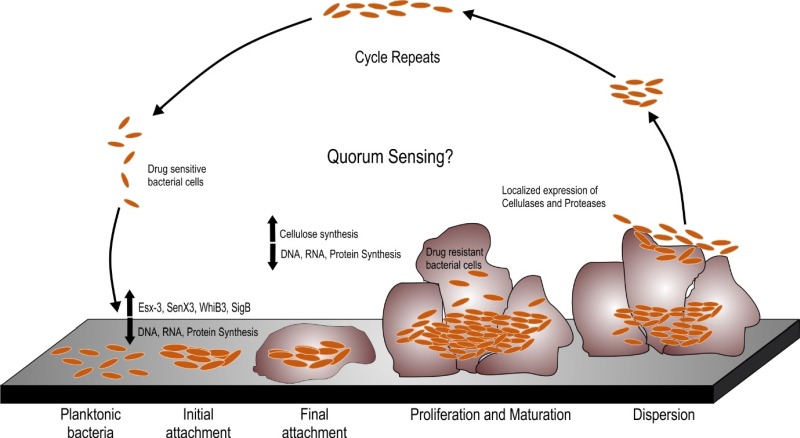
FIGURE 1: The biofilm formation and dispersal cycle. The planktonic bacteria form biofilms through a series of steps, which involve the initial attachment of the cells to a substratum followed by biofilm maturation and proliferation of bacteria within the matrix and finally a part of the matured biofilm dispersing to another site for subsequent localization and attachment. During this process, bacteria undergo phenotypic changes. Several genes playing roles in virulence and redox sensing are upregulated. Biofilms formation is associated with upregulation in cellulose synthesis during maturation of the biofilms; however, localized expression of cellulases and proteases degrades the extracellular the matrix of the biofilm thereby leading to bacterial dispersal followed by start of a new cycle of biofilm formation.

A number of Mycobacterial species are known to form biofilms including *Mycobacterium tuberculosis* (Mtb), *Mycobacterium smegmatis* (Msm), *Mycobacterium avium, Mycobacterium marinum* and *Mycobacterium ulcerans* [9-12]. Schulze *et al*. described the capability of Mycobacterium to form biofilms in 1989 [13]. In this pioneering work, they analyzed the capability of *Mycobacterium kansasii* and *Mycobacterium flavescens* to form biofilms in water drainage systems. They demonstrated densely packed mycobacterial colonies in the silicone tube constantly perfused by the water from the distribution system. The same group further reported the occurrence of ~ 4.5×10^5^ CFU/L of *Mycobacterium chelonae, Mycobacterium gordonae, Mycobacterium fortuitum, M. kansasii* and *M. flavescens* in the biofilms formed in domestic water supply systems using specific biochemical reactions and thin layer chromatography for mycolic acids [14, 15]. A number of studies have shown the presence of non-tuberculous mycobacteria (NTMs) in the cooling water distribution systems, dental sprays [16], potable drinking waters [17, 18], water filters in contaminated hospital bronchoscopes [19], and other environmental sources. These sources could act as a reservoir for NTMs that could infect humans and animals through swallowing, inhalation or inoculation and subsequent colonization in oral, respiratory or gastric wounds. In the last two decades, understanding the mycobacterial biofilms has evolved into a niche area for research. It must be noted that both pathogenic and non-pathogenic species of mycobacteria are capable of forming biofilms and this capability is not essentially a virulence mechanism. However, biofilms could protect the pathogenic mycobacterial species from the immune-system of the host and could help bacteria to persist during treatment with antibiotics. Given these observations, studying biofilms of pathogenic mycobacterial species is important. In this review, we will describe the pertinent information on mycobacterial biofilms with emphasis to their clinical relevance and the nature of EPS. The biofilm formation occurs through a series of steps involving the initial attachment of the bacterial cells to substratum which is followed by the aggregation of the cells and irreversible binding. This step is followed by maturation of the biofilm cells which is formed by layering of the aggregates, which upon reaching an ultimate thickness starts to disperse only to start aggregating at a new site (as depicted in Figure 1). Current understanding of the mechanisms and characteristic features of mycobacterial biofilms are described in this review.

## BIOFILM FORMATION BY *MYCOBACTERIUM SMEGMATIS*

Msm is a rapidly growing non-pathogenic mycobacterial species that is often used as a model organism for studying the mycobacterial physiology [20]. Since it is a model organism, it is quite well studied for the biofilm formation. Msm is known to form well organized colonies and microcolonies which have been described by Danese *et al*. as a type of biofilm. These colonies are composed of microbial cells encapsulated by a large amount of exopolysaccharides [21]. Importantly, sliding motility plays a critical role in the formation of colonies on plate [22]. Recht *et al*. demonstrated that transposon mutants in the glycopeptidolipids (GPL) biosynthesis pathway are attenuated for colony formation and lack the capability to form biofilms on PVC plates [22]. GPLs are an important component of the mycobacterial cell wall, and it was observed that they play an important role in the initial attachment of mycobacterial cells to the substratum like PVC. The same group later on demonstrated that acetylation of GPL is also important in determining the colony morphology and formation of biofilms on PVC plates [23]. Subsequently, mycolic acids, another major component of the mycobacterial cell wall, was implicated in pellicle biofilm formation. Pellicle is a bacterial growth at the media-air interface. This mode of growth is primarily seen in aerobic bacteria wherein the bacterial cells have access to both air and nutrients of media. Some of the recent studies have demonstrated that in the pellicle, the mycobacterial cells are encapsulated in self-produced EPS [24]. Thus, mycobacterial pellicles are considered to be a form of biofilms. In an exciting discovery, Ojha *et al*. demonstrated that mycolic acids play a critical role in maturation of pellicle biofilms [25]. Importantly, the mycolic acids produced during maturation of pellicle growth are shorter (C_56_-C_68_) compared to the regular mycolic acids of the cell wall of Mycobacteria (C_70_-C_70_). Apparently, chaperone GroEL1 regulates this transition in the type of mycolic acid and thus plays an important role in pellicle biofilms formation [25]. Ojha and colleagues also suggested that the short chain free mycolic acids are released through hydrolysis of trehalose dimycolate (TDM) by serine esterase [26]. Interestingly, Msm *rpoZ* gene (encoding for the ω subunit of the RNA polymerase), deletion mutant displayed an altered colony morphology. Analysis of this mutant revealed that it is defective in sliding motility and biofilm formation [27]. Importantly, this mutant has equal quantities of GPL compared with wild type Msm. Mass spectroscopy-based analysis of mycolic acids suggested that it possessed very low levels of short chain mycolic acids. SEM analysis demonstrated the absence of ECM in the pellicle growth of the mutant. These findings strongly support that short chain mycolic acids are a component of ECM [27]. The role of lipids in biofilm formation is also supported by the observation that a Msm mutant in *mmpL11* (required for the transport of monomeromycolyl diacylglycerol (MMDAG) and mycolate ester wax to the bacterial surface) has a delay in biofilm formation [28]. However, it must be noted that this mutant forms quite mature pellicle biofilms sometime later. The role of MMDAG in cell-to cell attachment and biofilm formation was also independently demonstrated in a separate study [29]. In this study a transposon mutant library was created and analyzed for defects in colony morphology. It was observed that transposon insertion in the Lsr2 (a histone like protein in mycobacteria) leads to smooth, wet, and round colonies, opposed to the dry, rough, and rugose colonies of the parent Msm strain. This strain was also compromised in biofilm formation. Further analysis suggested that this transposon mutant strain has equivalent levels of GPLs and mycolic acid, but is deficient in the MMDAGs [30]. It was further suggested that the presence of MMDAGs on the cell surface may increase its hydrophobicity to facilitate cell-to-cell interaction. The role of Lsr2 in biofilm formation was confirmed by another study, which demonstrated that Lsr2 mutant of Msm has increased sliding motility, reduced surface hydrophobicity and is attenuated for pellicle growth [31]. The role of Lsr2 in the different genetically programmed stages of biofilm formation in Msm was further illustrated by Yang *et al*. in 2017, where they monitored the participation of different genes in the individual stages of biofilm formation – from attachment with the substratum to intercellular aggregation followed by maturation of the pellicle architecture [32]. The group presented a model of biofilm formation where the planktonic cells, with the help of Lsr2, start forming aggregates which in turn triggers upregulation of GroEL and GroEL dependent free mycolate synthesis. It is worth noting that aggregation may impart benefits like drug tolerance, but it is not equivalent of biofilms. However, it shall be noted that bacterial aggregates may play a role in biofilm formation in bacterial species such as *P. aeruginosa*, and that variant strains that readily make aggregates also form strongly adherent biofilms [33-35]. It further induces iron sequestration pathways, which mark the onset of biofilm maturation [32]. The role of the cell wall in pellicle biofilm formation is also supported by the observation that the Δ*hadC* mutant with a defect in dehydratase activity of fatty acid synthase type II (FAS-II) takes longer time for pellicle formation and has altered colony morphology [36]. On the contrary, a mutant of the mammalian cell entry (mce) 1 operon that accumulates free mycolic acids in its cell wall forms normal colonies [37]. Whether these mutants form better pellicle or make it rapidly remain to be analyzed. However, the *mce* operon mutant of Msm, wherein all the six operons are deleted, is attenuated for pellicle formation and has altered colony morphology [38]. It will be interesting to see if this mutant also accumulates free mycolic acids in the cell wall. Intriguingly, ectopic expression of a putative peptidoglycan amidase (Rv0024) in Msm induces biofilm formation and leads to an increase in drug resistance [39]. Importantly, overexpression of Rv0024 was associated with increased hydrophobicity of mycobacterial cells. These observations suggest that abundant amounts of free mycolic acids may still be the part of the cell wall, but lead to increased surface hydrophobicity that may help the mycobacterial cells to associate more with each other. However, if this hypothesis is true, then the free mycolic acids are not really part of the extracellular matrix (since they are part of the cell wall, rather than the extracellular matrix). This hypothesis is supported by the observation that incubation of Msm cells with secretory antigen MTC 28 (encoded by Rv0040c) increases hydrophobicity of the mycobacterial cells and induces cellular aggregation [40]. Furthermore, inhibition of peptidoglycan biosynthesis by knockdown of phosphoglucosamine mutase (*glmM*) also reduces biofilm formation [41]. These observations suggest that composition of the cell wall could greatly affect the mycobacterial biofilm formation through modulation of initial cell-to-cell interaction. We believe that further research is required to study the effect of changes in cell-to-cell contact on the biofilm formation.

How does the mycobacterial biofilms form and what induces their formation have remained important questions in the field. In the recent years, answers to these questions are emerging, but the entire picture is not as clear as is desired. A number of redox stresses are known in other bacteria to induce biofilms [42]. Bhat *et al*. have earlier demonstrated that redox stress in culture and/or inside macrophages leads to accumulation of NADH levels in mycobacterial cells [43, 44]. Interestingly, intracellular NADH levels are sensed by the PknG. PknG, along with ribosomal proteins L13 and Nudix hydrolase RenU, constitutes a redox homeostatic system responsive to cellular NADH levels named as RHOCS. Wolff *et al*. demonstrated that upon sensing higher cellular levels of NADH, PknG phosphorylates L13 protein and thus increases its association with RenU. L13 with RenU leads to NADH hydrolysis thereby balancing redox homeostasis in the cells. Interestingly, PknG, L13 and RenU all are required for biofilm formation by Msm [45]. These findings suggest that the metabolic state of the mycobacterial cells regulates the biofilm formation in Mycobacteria. On one hand, the NADH:NAD^+^ redox couple along with the ATP:ADP depicts the metabolic state of the cell, on the other hand, mycothiol along with the antioxidant ergothioneine constitutes the thiol buffering system of the mycobacterial cells [46]. Interestingly, Msm mutants in mycothiol biosynthesis (Δ*mshC*) or mycothiol dependent metabolism of nitrosothiols (Δ*mscR*) are compromised for pellicle biofilm formation [47]. These observations suggest a critical role of the redox state in the biofilm formation. Along these lines, Trivedi *et al*. demonstrated that intracellular thiol reductive stress induces biofilm formation in Mtb cells. It remains to be analyzed whether thiol reductive stress also induces biofilm formation in Msm or other mycobacterial species. Besides the redox stress, marker of the stringent stress response (p)ppGpp and cyclic nucleotide c-di-GMP play a critical role in biofilm formation. (p)ppGpp is synthesized by Rel_Msm_ while the c-di-GMP is synthesized by DcpA. Msm knockout strains of these second messengers (Δ*relA* and ∆*dcpA*) are compromised for biofilm formation [48]. Furthermore, Kuldeep *et al*. have demonstrated that low levels of these second messengers assist in bacterial growth, while higher intracellular concentration promotes biofilm formation [49]. However, the mechanisms through which the altered redox and metabolic state modulates these second messengers to promote biofilm formation needs to be delineated.

It shall be noted that biofilm formation is an active process that is tightly regulated at translational and transcriptional levels. In order to understand the genes involved in biofilm formation, Ojha *et al*. studied the transcription profiling of Msm biofilms [50]. They demonstrated that a 3-day old Msm biofilm had 1.5% differentially regulated genes, whereas 4.5% of the total genes are modulated in the 4-day old biofilm and 4.9% in the stationary phase Msm cultures. There was an increase in the expression of mycobactin biosynthesis genes, exochelin biosynthetic genes and the putative iron ABC transporter in the 3-day and 4-day old biofilm cultures, suggesting an importance of iron uptake in the development of Msm biofilms. But more transcriptomics experiments need to be performed to generate a transcriptional map of the important regulatory network that plays an important role in the biofilm formation.

## BIOFILM FORMATION BY THE NON-TUBERCULOUS MYCOBACTERIA (NTMS)

NTMs include all the mycobacterial species (~178 different species listed at http://www.bacterio.net/mycobacterium.html) besides the ones classified under the ”*Mycobacterium tuberculosis complex”* and those known to cause leprosy (*Mycobacterium leprae*). These are also known as ”mycobacteria other than tuberculosis” and ”atypical mycobacteria” [17]. NTMs are ubiquitous and are found in diverse environments such as soil and water. These mycobacterial species could infect and cause skin and soft tissue infections (SSTIs) in animals and humans [17]. A number of the NTMs make biofilms naturally in the environment [51]. NTMs such as species belonging to the *Mycobacterium avium* complex form biofilms [12] and also cause infections in humans and animals [52]. Given that many of the NTM infections are chronic and require long treatment, studying their biofilms could be relevant to reduce the course of treatment. Although a number of NTMs form biofilms in the environment, here we have focused on the biofilms of medically relevant species of NTM.

### Mycobacterium avium

*M. avium* is capable of infecting humans [53], domestic animals [54] and birds [55] and is found abundantly in different environmental niches such as water bodies and soil. Being an opportunistic pathogen, it mostly infects immunocompromised patients, especially those suffering from AIDS [53], cystic fibrosis [56], or pulmonary alveolar proteinosis [57]. Falkinham *et al*. demonstrated the presence of *M. avium* biofilms in the drinking water distribution system [58]. Following that, many studies have confirmed that *M. avium* forms detectable biofilms in potable drinking water as well as household plumbing water pipes [59-61]. Importantly, several human, avian and porcine isolates of *M. avium* are capable of forming biofilms *in vitro* when incubated for 7 days as a suspension in 7H9 medium with O-ADC and Tween in the 96-well flat bottom polystyrene microtiter plate [62]. *M. avium* infections are difficult to treat and require prolonged treatment with multiple drugs. Formation of *M. avium* biofilms *in vivo* could explain the requirement of such a prolonged treatment. Formation of colony biofilms has been studied extensively for *M. avium*. Sliding motility is dependent on the presence of glycopeptidolipids (or GPLs are a class of amphiphilic molecules localized in the cell envelope) and plays a critical role in colony biofilm formation in *M. avium* [22, 63]. Interestingly, Yamazaki *et al*. observed a correlation between *M. avium* biofilm formation and its capability to colonize the bronchial and bronchiolar mucosa [64]. *M. avium* strains, namely MAC104, MAC101 and MAC A5 could invade and infect the bronchiolar epithelial cells. Incidentally, these strains are also capable of forming biofilms *in vitro*. However isogenic mutant clones of MAC A5 (namely 5G4, 6H9 and 9B5), that are attenuated to form biofilms were unable to invade and infect the bronchiolar epithelial cells. Additionally, it was observed that these mutant strains were also compromised in their ability to infect mice lung while the MAC A5 was capable of infecting mice lungs, spleen and liver. Furthermore, *M. avium* biofilms or their supernatants were capable of inducing TNF-α production when compared with their planktonic counterparts. This excessive TNF-α production in response to *M. avium* biofilms leads to apoptotic cell death of the macrophages [65]. Investigations on biofilm formation suggested a requirement of divalent cations like Ca^2+^, Mg^2+^, Zn^2+^ for biofilm formation [12]. These might act as stabilizing agents for the negatively charged nucleic acids present in the biofilms. Besides these agents, the presence of glucose and peptone as carbon sources enhanced the biofilm formation. On the contrary, humic acid could inhibit biofilm formation. Interestingly, the supernatant from the *M. avium* biofilm culture induced biofilm formation in planktonic cells suggesting some kind of quorum sensing could assist biofilm formation [12]. Also, oxidative stress induced by Autoinducer-2 (AI-2) activates biofilm formation in *M. avium* [66]. Apart from GPLs, extracellular DNA (eDNA) has also been found to be a part of the *M. avium* biofilm matrix. Importantly, exposure to Dnase disrupts *M. avium* biofilms suggesting that DNA is an integral component of the EPS of *M. avium* biofilms [67]. Generally, eDNA is produced in biofilms through (i) autolysis, (ii) active secretion, (iii) and via membrane vesicles [68]. The release of eDNA is governed through quorum sensing. Interestingly, an unbiased transposon mutant screening identified the FtsK/SpoIIIE DNA transport system and carbonic anhydrase as sufficient components for DNA export in *M. avium*, and these genes were induced by bicarbonate [69]. These observations point to a quorum sensing based mechanism for production of eDNA in *M. avium* biofilms. Another study identified 6-oxodehydrogenase (*sucA*), enzymes of the TCA cycle, protein synthetase (*pstB*), enzymes of glycopeptidolipid (GPL) synthesis, and Rv1565c (a hypothetical membrane protein) to play an important role in biofilm formation [70]. However, a detailed analysis of the extracellular polymeric substance of *M. avium* biofilms remains to be done.

### Mycobacterium abscessus

*M. abscessus* (Mab) is a fast-growing NTM that causes a wide variety of human infections, including those of lung, skin, soft tissue, ocular and central nervous system, etc. These infections are recalcitrant to treatment with a multitude of antibacterial drugs [71]. Mab is emerging as a major pathogen associated with cystic fibrosis [72]. In an elegant study, Qvist *et al*. demonstrated the presence of Mab microcolonies surrounded by extracellular matrix in sparse intra-alveolar walls [73]. Microcolonies are microscopic communities of ~50 cells that spontaneously aggregate and could nucleate the growth of a biofilm. In the above-mentioned study, the aggregates/microcolonies were mostly observed embedded deep in the alveolar wall and only occasionally observed in the phagocytosed Mabs [73]. Importantly, these microcolonies were equated to biofilms in the absence of any evidence for the self-produced extracellular matrix. In our view, more studies are required to demonstrate the presence of extracellular matrix surrounding the bacterial communities to conclude the presence of mycobacterial biofilms in the lungs. Such studies could prove to be milestones in the current understanding of the pathogenesis of diseases caused by mycobacteria. Interestingly, another study by Fennelly *et al*. demonstrated the presence of Mab biofilms in the lung cavity of a patient with chronic obstructive pulmonary disease using *M. abscessus* specific PNA-FISH probes. They also demonstrated that ~2% of Mabs in the lung cavity were in the biofilms and were embedded in the extracellular matrix [74]. Interestingly, bacteria residing in biofilms exhibit drug tolerance [75]. A few other reports also suggest that Mab biofilms formed on implants can lead to post-operative surgical site contamination [76, 77]. It is worth mentioning that Mab grows on LJ slants and Middlebrook 7H10 agar to produce two different colony morphologies, namely rough (Mab-R) and smooth (Mab-S). Importantly, rough and smooth morphotypes exhibit different virulence phenotypes [11]. Interestingly, Mab-R forms extensively corded microcolonies and is virulent, on the other hand, Mab-S forms rounded, smooth, smaller microcolonies and is less virulent [11, 78]. Further studies on the morphology switching revealed that the gene *mab_3168c*, encoding for a putative acetyltransferase, regulates this morphotypic switching and the *mab_3168c* deletion mutant grows in smooth colonies. Importantly, Δ*mab_3168c* is unable to revert back to rough colonies and is deficient in biofilm formation and intracellular survival [79]. Although much information is not available about the nature of EPS of this NTM, few reports suggest that the matrix of Mab biofilms contains glycopeptidolipids as well as extracellular DNA [67]. The secretion of eDNA is regulated by bicarbonate and is important for structural integrity of the biofilm [69]. The role of glycopeptidolipids in Mab biofilms has been worked out using genetic approaches. It was observed that deletion of genes like *mmpL4b* [80] or *mab_3168c* [79], that play important roles in the glycopeptidolipid biosynthetic pathway, results in impaired biofilm formation. However, more studies are required for characterization of the nature of extracellular matrix of this pathogen.

### *Mycobacterium fortuitum* and *Mycobacterium chelonae*

Other important fast-growing human pathogens are *M. fortuitum* and *M. chelonae*. These are opportunistic human pathogens that primarily infect people with compromised immune system or those suffering with chronic diseases [81]. These species are also known to lead to post-surgical infections and could form biofilms in eye or skin tissues [82]. These species quickly form biofilms within 48 hrs [83]. The biofilm formation is robust and does not depend upon the nature of the substrate [84]. Importantly, these biofilms are biocide resistant [85]. The biofilms of *M. fortuitum* are impermeable to several antibiotics, including ciprofloxacin, thereby suggesting that biofilm permeability of antibiotics might be an important reason behind antimicrobial drug resistance. Interestingly, these biofilms contain eDNA and a combination of antibiotics with DNase was more effective in disrupting the biofilms and killing biofilm residents than antibiotics alone [9].

### Mycobacterium ulcerans

*M. ulcerans* causes buruli ulcer in humans. Buruli ulcer is the third most common infection caused by a mycobacterial pathogen after tuberculosis and leprosy. These infections are most commonly reported from sub-saharan Africa [86]. A unique feature of *M. ulcerans* infection is the development of necrotic cutaneous lesions caused by polyketide toxin – Mycolactone [87]. Importantly, Mycolactone is among only few of the virulence toxins identified for mycobacterial species. *M. ulcerans* is a slow-growing environmental bacterium that is capable of forming *in vivo* biofilms on the salivary glands of the aquatic insect *Naucoris cimicoides* [88] and on aquatic plants [89]. In an elegant study, Marsollier *et al*. [90] characterized the biofilms of *M. ulcerans*. SEM analysis also revealed that in the biopsy samples from Buruli ulcers patients and those isolated from infected mice mycobacterial cells were organized into discrete bacterial clusters enveloped in the extracellular matrix suggesting the formation of biofilms *in vivo*. Importantly, these biofilm-like structures also contained vesicles between 50 and 200 nm in diameter. Similar vesicles were also detected in the ECM from *M. ulcerans* biofilms. They further showed that these vesicles contain Mycolactone and its biosynthesis machinery. This group was able to separate the ECM from the bacterial cells using mechanical disruption (with glass beads) along with treatment with Tween 80 detergent. They demonstrated that the ECM was capable of protecting the mycobacterial cells from antimycobacterials such as Rifampicin. They further demonstrated that more than 80 proteins are present in the ECM and that these proteins play roles in stress responses, respiration and intermediary metabolism. They also revealed that *M. ulcerans* biofilms are rich in carbohydrates, with glucose being the most abundant monosaccharide unit, relating it structurally to the D-glucan of Mtb. More research work is required to fully understand the role of polysaccharides in the ECM of *M. ulcerans* biofilms. Other ECM components include lipids such as phosphatidylinositol mannosides (PIM2, PIM5 and PIM6), phospholipids (phosphatidylethanolamine, phosphatidylinositol, cardiolipin), triacylglycerol, phthiodiolone diphthioceranates, etc [78]. The bacterial adherence and attachment to the surface are enhanced by the small 18-kDa-heat shock protein (Hsp18) [91] suggesting an important role for this chaperon in the biofilm formation.

### Mycobacterium marinum

*M. marinum* is a slow-growing bacterium that causes infection in fish and occasionally infects humans. Its infection in Zebrafish has been used as an important model system for teasing out the molecular events associated with TB pathogenesis [92]. Importantly, *M. marinum* could form biofilms within 14 days on a variety of abiotic surfaces. However, silicon surface yielded the highest levels of biofilm production. Hall-Stoodly also studied the ultra-structure of these biofilms and observed that the cording of mycobacterial cells progressed during the later phase of biofilm development [93]. This cording phenotype was suppressed by OADC supplement [93]. Lipooligosaccharides are cell wall components and play an important role in cell motility. *M. marinum* mutants incapable of forming lipooligosaccharides were defective in biofilm formation [94]. The role of phthiocerol dimycocerosates (PDIMs) and phenolic glycolipids (PGLs) in biofilm formation by *M. marinum* was analyzed by Mohandas *et al*. [95]. They demonstrated that genetic mutants defective in the PDIM/PGL biosynthetic pathway are attenuated for biofilm formation. These mutations also affect cell-surface properties but not sliding motility. These mutants also display increased antibiotic susceptibility [95]. However, the precise nature of the extracellular matrix of *M. marinum* biofilm remains to be deciphered.

## BIOFILMS OF *MYCOBACTERIUM TUBERCULOSIS*

Mtb causes tuberculosis (TB) and is the leading cause of human deaths due to a single pathogen. The currently used treatment of TB involves usage of multiple drugs namely isoniazid, rifampicin, pyrazinamide, streptomycin and ethambutol for at least 6-9 months. Such lengthy treatment results in non-compliance and emergence of multi-drug-resistant TB (MDR-TB) and extensively drug resistant tuberculosis (XDR-TB). However, it must be emphasized that such a combination can efficiently eliminate Mtb in culture in significantly shorter duration of time. These differences in the killing of Mtb cells under laboratory conditions and in infected hosts point towards a disconnect between the current understandings of Mtb physiology emerging from labs and its actual physiological state in humans. Current literature suggests two plausible hypotheses to explain the phenotypic drug tolerance displayed by the genetically drug susceptible Mtb inside the host. The first hypothesis suggests that Mtb senses the host environment and a fraction of Mtb cells transits into non-replicating persistent (NRP) state. During the NRP state, Mtb cells are believed to be metabolically quiescent, but generating sufficient energy to keep the membrane energized [96, 97]. Since most of the antimycobacterial drugs (besides bedaquiline) target components of active cell growth, these metabolically quiescent cells are drug tolerant. It is well documented that the NRP state could be induced by hypoxia, nitric oxide and starvation [46, 98-101] and could be reversed by resumption of ambient oxygen and nutrients, declining nitric oxide levels [102]. Another hypothesis to explain the phenotypic drug tolerance is the formation of Mtb biofilms inside the host. Mtb forms biofilms harbouring drug-tolerant bacteria *in vitro* [10, 103] however, the factors controlling the biofilm formation and the properties of the extracellular material are poorly known. The Mtb biofilm hypothesis arose from the work of Ojha *et al*., wherein Mtb pellicle growth was equated to the biofilms as pellicles contain selfproduced EPS which holds the cells together. Interestingly, this study also demonstrated that Mtb cells residing in the pellicle exhibit drug tolerance and harbour significantly higher number of persister Mtb cells. In the following section, we have described the most pertinent information regarding the Mtb biofilms.

### Mtb biofilm models

**Figure 2:**
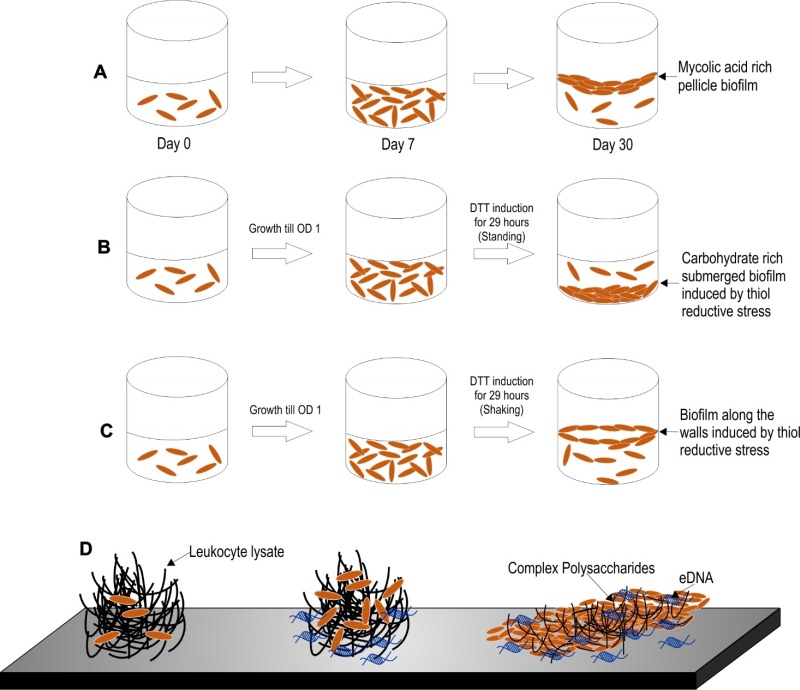
FIGURE 2: Different models of biofilm formation in *M. tuberculosis*. **(A)** *Pellicle biofilm model of M. tuberculosis*. The pellicle biofilm matures through several stages of development in around 5-7 weeks. These biofilms are rich in free mycolic acids. **(B-C)** *Thiol reductive stress induced biofilm of M. tuberculosis*. This model is induced by thiol reductive stress generated by reduced DTT. This polysaccharide rich biofilm of Mtb takes around 29 hours to develop. Keeping the culture flask at standing position generates a biofilm that attaches to the bottom surface of the flask **(B)**, whereas shaking of the culture leads to biofilm formation at the liquid-air interface **(C)**. **(D)** *Leukocyte lysate induced biofilm model of M. tuberculosis*. This eDNA rich biofilm of Mtb takes around 7 days to develop. This model may depict the biofilms formed inside the granuloma, wherein leukocyte lysate is available due to cell lysis induced by Mtb cells.

Three models of Mtb biofilm formation have been proposed to study the factors regulating biofilm formation, the physiology of the resident bacteria, and the nature of biomaterial that holds these bacterial masses together. These models include pellicle biofilms formed at the liquidair interface of cultures, leukocyte lysate-induced biofilms, and thiol reductive stress-induced biofilms (depicted in the Figure 2).

### Pellicle biofilms

Mtb has a propensity to grow as pellicles at the liquid-air interface *in vitro* cultures. The Mtb pellicles are formed through several stages of development over a period of 5-7 weeks [104]. Keto-mycolic acids play an important role in pellicle formation and has been proposed to be a structural component of ECM in pellicle biofilms [24]. The usage of this model of biofilms for the screening of antimycobacterial compounds resulted in the identification of the potential antimycobacterial drug candidate TCA1 [105]. Major advantage of the pellicle model of Mtb biofilms is that it allows for tracking of the different developmental stages during biofilm maturation. Given the simplicity of this model, it remains one of the most studied models of Mtb biofilm formation.

### Leukocyte lysate-induced biofilms

Recently, Ackart *et al*. demonstrated that mycobacterial cells organize into substratum-attached drug tolerant microbial communities in culture media (RPMI 1640) supplemented with the lysate of leukocytes over a period of 7 days [106]. Importantly, the drug tolerant phenotype of these mycobacterial communities could be reverted through the disintegration of the communities, using DNase or Tween 80. These studies clearly depict that the drug tolerance of mycobacteria could be explained solely through their capability to form biofilms. It is important to note that inside caseous necrotic granulomas, extracellular mycobacteria are exposed to leucocyte lysate. Thus, this model, in a way mimics the *in vivo* environment. Intriguingly, this model was employed in the discovery of molecules capable of dispersing Mtb communities and, thus, aiding killing by first-line anti-TB drugs [107]. Based on these findings, the classical anti-TB therapy could be shortened in the future through the use of biofilm-dispersing adjunct therapy with similar anti-biofilm agents.

### Thiol reductive stress-induced biofilms

Recently, another model of Mtb biofilms was established by Kumar and coworkers [108]. In this model, upon exposure to thiol-reductive stress (TRS), Mtb cells organize into microbial communities strongly attached to the substratum. The architecture of the microbial community depends on the prevailing culture conditions, i.e., submerged biofilms form in standing cultures while biofilms at the liquidair interface form in shake flask cultures. One of the biggest advantages of this model is that these biofilms require only 29-30 hrs for biofilm formation in comparison to the 7 days required for biofilms induced by host cell-derived complex macromolecules and ~35 days required for the development of pellicle biofilms [108]. Owing to the short duration required for biofilm formation in this model, the processes of bacterial cell attachment, cellular differentiation and the synthesis of EPS are amenable to tracking through microscopy, transcriptomic, proteomic and metabolomic profiling. Furthermore, these biofilms are strongly attached to the substratum similar to conventional biofilms, such that a simple treatment with the detergent Tween 80 or manual shaking does not disrupt these biofilms.

## TRANSCRIPTIONAL CHANGES ASSOCIATED WITH BIOFILM FORMATION

In order to understand the tight regulation of genes at the transcriptional levels in TRS induced biofilm formation, Trivedi *et al*. analyzed the transcription kinetics, both when the Mtb cells were subjected to sub-optimal TRS as well as optimal TRS for biofilm formation. Upon milder treatment, the genes involved in protein synthesis are downregulated suggesting that the cells go into energy conserving mode. Genes responsible for iron uptake, aerobic respiration and lipid degradation are, however, upregulated. Upon treatment leading to high TRS, DNA replication, RNA biosynthesis as well as protein synthesis machinery come to a halt, as all the genes involved in these central processes are downregulated, which is again suggestive of the fact that the bacterial cells stop replicating. The formation of TRS-induced Mtb biofilms is associated with induction of *sigE, sigB* and *whiB3* expression. However, the precise role of *sigE, sigB* and *whiB3* in the formation of mycobacterial biofilms remains to be defined. Importantly, the type VII secretion system ESX-3 is upregulated in the Mtb biofilm which indicates a requirement of iron uptake in biofilm formation. In agreement *furA*, responsible for iron uptake, is highly upregulated in Mtb biofilms. Other upregulated genes involve those playing a role in cysteine and arginine metabolism. It must also be noted that the SenX3/RegX3 system which is induced upon mild TRS is also overexpressed in the Mtb cells residing in the biofilms [108]. The SenX3/RegX3 two component system is involved in growth in response to resumption of ambient oxygen levels [102]. These data suggest that oxygen is not evenly distributed in biofilms. Interestingly, PknA is also induced in the biofilm resident Mtb cells. Since PknA is involved in the maintenance of bacterial cell growth [109], these data suggest that some bacterial cells may be growing in the biofilm. However that the transcriptomic data obtained through microarray experiments do not reflect upon the differential expression of genes in the bacterial cells localized at different spatial location in the biofilms and hence such data should be analyzed with caution. We believe that development of tools having spatial resolution for monitoring gene expression could shed light on the differential expression in biofilms.

## PHENOTYPIC DRUG TOLERANCE IN BIOFILMS

Phenotypic drug tolerance is the ability of genetically drug susceptible bacterial cells to evade killing by the antimicrobial agents [110]. It is worth noting that Mtb biofilms, developed using either of the three models, harbor phenotypically drug-tolerant Mtb cells. However, the mechanisms and molecular events that dictate the drug tolerance of the biofilm-resident cells have not been deciphered. Several mechanisms have been proposed to explain the phenotypic drug tolerance displayed by biofilm-resident bacteria [111], i.e., metabolic heterogeneity of biofilm residents, increased persister population, induction of reactive oxygen species scavengers, increased expression of efflux pumps, extracellular drug inactivation, protection by polysaccharides that acts as a physical barrier resulting in low penetrance of antibiotics, etc. Currently, we don't know which of these mechanism/s contribute more for the phenotypic drug tolerance but the study from Ackart *et al*. and Trivedi *et al*. suggest that the formation of bacterial communities is important for the observed phenotypic drug tolerance [106, 108]. Understanding such mechanisms for Mtb will be helpful in designing new therapeutic agents that may reduce the duration of TB therapy. In this direction, Ojha *et al*. demonstrated that the pellicle biofilms of Mtb harbor a significantly larger number of persister cells compared to planktonic cells [104]. It is commonly believed that bacterial cells residing in the biofilms are metabolically quiescent and, thus, display drug tolerance. However, Trivedi *et al*. reported that the ATP/ADP, NADH/NAD^+^, NADPH/NADP^+^ ratios of Mtb cells residing in TRS-induced biofilms were only slightly lower than those of planktonic bacteria [108]. However, such studies presume that the metabolic states of all residents of the biofilm are similar while bacterial cells in different regions of the biofilms have differences in terms of access to nutrients and thus are expected to have a different metabolic state. To dissect the metabolic heterogeneity of biofilms resident bacteria, new tools with spatial resolution (and preferably temporal resolution as well) are urgently required. Recently, genetically encoded biosensors for the measurement of the metabolic [43, 44], energy [112] and redox state [113] of Mtb cells were developed. The application of these biosensors to understand the metabolic flux and redox state of the residents of Mtb biofilms represents a new research opportunity. Furthermore, knowing absolute concentration of the metabolites with spatiotemporal details will be desirable. It is worth noting that alterations of the redox homeostatic system that controls the cellular levels of FADH and NADH modulate biofilm formation [45]. Importantly, accumulation of intracellular thiol also facilitates the formation of Mtb biofilms [98]. Redox homeostasis is also known to play an important role in the persistence of Mtb [98, 99]. Furthermore, Trivedi *et al*. performed transcriptome kinetic analysis during the biofilm formation. This analysis suggested that biofilm-resident bacterial cells utilize alternative metabolic pathways for the generation of energy [108]. However, we believe that such a transcriptomic analysis only provides a bird's eye view of the transcriptional changes associated with the biofilms. New tools having spatiotemporal resolution could provide details of the differential transcriptional regulation in different regions of the biofilms are needed to understand the transcriptional profile of biofilm resident bacteria. Further analysis of metabolic profiles, using other techniques such as quantitative mass spectroscopy, could help us to understand the metabolic networks that play critical roles in the biofilm formation, maintenance, and disruption. Once these alternative metabolic pathways are delineated, they could be targeted to kill the biofilm-resident mycobacterial cells.

### Extracellular matrix of biofilms

**Figure 3:**
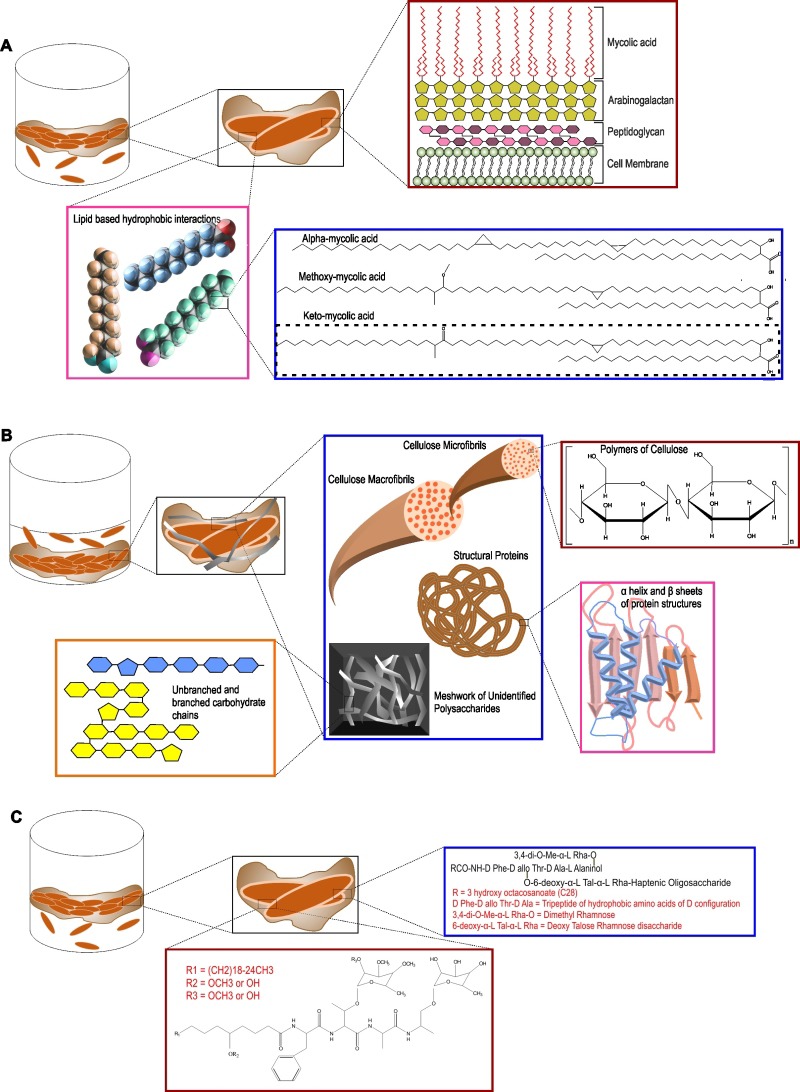
FIGURE 3: Different components of the extracellular polymeric substance of mycobacterial biofilms. **(A)** *The pellicle biofilm model*. The inset in maroon shows the different components of the mycobacterial cell wall. The inset in pink shows the lipid based hydrophobic interactions that hold the cells in the biofilm together. The inset in blue shows the different types of mycolic acids present in the extracellular biofilm matrix, the most abundant being keto-mycolic acid. **(B)** *The thiol reductive stress induced submerged biofilm model of Mtb*. The inset in blue shows the presence of polysaccharides, cellulose macro- and micro-fibrils, different structural proteins and meshwork of other unidentified branched and unbranched polysaccharides. **(C)** *The pellicle biofilm model of M. smegmatis and M. avium*. Apart from mycolic acids, glycopeptidolipids (GPLs) play a major role in pellicle biofilm formation in these bacteria. The inset in maroon depicts the GPL structure in *M. smegmatis* and the same in blue depicts the GPL structure in *M. avium*.

The extracellular matrix consists of EPS, produced by cells present in the biofilm. Self-produced EPS is also considered to be the hallmark of a microbial biofilm [114]. Bacterial cells, along with nutrients and enzymes, are embedded in this EPS. EPS acts as the glue that keeps the bacterial cells together in microcolonies and attaches them to the substratum. EPS allows cell to cell interaction, communication and synergy within the microcolonies [114]. EPS represents a wide array of polymers, including proteins, nucleic acids, polysaccharides and lipids that serve as carbon and energy reserves. EPS could also be accumulated on the cell surface to protect the cells against the external environment. In most types of microbial biofilms, the EPS is composed of polysaccharides, structural proteins, extracellular DNA (eDNA), and lipids (as depicted in Figure 3). In the following section, we have described each of the components of the extracellular matrix of Mtb biofilms.

### Lipids

One of the most studied models of Mtb biofilms are pellicle biofilms. Importantly, mycobacterial pellicles contain large quantities of free mycolic acids [25, 104]. This free mycolic acid is produced by cleavage of trehalose dimycolate using a TDM specific esterase [26]. An inability in keto-mycolic acid biosynthesis or in cleaving TDM leads to the inability to form pellicle biofilms or retarded biofilm growth [24]. Besides mycolic acids, meromycolyl diacylglycerol [29, 30] and glycopeptidolipids [23] also contribute to biofilm formation in mycobacteria. These studies suggested that mycobacterial biofilms are uniquely held together by a waxy EPS [115]. However, another hypothesis is that these cell wall components increase the cell-surface hydrophobicity to increase the cell-to-cell interaction and thus are required for biofilm formation, but are not the components of the EPS of Mtb biofilms.

### Polysaccharides

The view that mycolic acids/lipids are primary components of Mtb biofilms was recently challenged by the observation that large quantities of polysaccharides are present in TRS-induced Mtb biofilms [108]. Importantly, by staining with carbohydrate-specific stains, Texas Red, lectin Concanavalin A, and Calcofluor-white, this group of researchers demonstrated that polysaccharides are present in the extracellular space of the biofilm. Using Nile red to stain lipids, they also suggested that lipids primarily localize on mycobacterial cells and not in the extracellular space of the Mtb biofilms. This observation is also suggestive of mycolic acids being a component of the cell wall rather than EPS. On the other hand, polysaccharides were detected at the base of the microcolony stalk and between the microcolonies. These findings suggested that polysaccharides are the major component of the EPS. Basaraba and coworkers have previously demonstrated the presence of complex polysaccharides in the leukocyte lysate-induced substratum-attached biofilms of Mtb [106]. The presence of extracellular polysaccharides in mycobacterial biofilms is also supported by studies in which abundant Texas Red staining was observed in the extracellular matrix of *M. ulcerans* biofilms [90] and the observation that aggregation of the mycobacterial cell is influenced by sugars [116]. Importantly, Kumar and coworkers have further characterized these polysaccharides by several biochemical methods and demonstrated that TRS-induced Mtb biofilms employ cellulose as a major structural component [108]. Intriguingly, cellulose was detected primarily in the spaces between the microcolonies and some cellulose was also detected at the stalks of the microcolonies. Cellulose is a polymer of glucose linked through β(1→4)-glycosidic bonds. It is hydrophilic, but water-insoluble and has strength comparable or more to that of steel [117]. Given the strength, cellulose has been shown to be an important component of several bacterial biofilms [114, 118]. Importantly, cellulase can disintegrate TRS-induced Mtb biofilms, suggesting that cellulose is critical for Mtb biofilm formation. The role of cellulose as an integral component is also supported by the observation the Mtb genome encodes for cellulases [119, 120] that could facilitate the dispersal of Mtb biofilms. The role of cellulase in mycobacterial pellicle biofilms was also analyzed by Wyk *et al*. [121], through over-expression of Rv0062 homologue MSMEG_6752 in Msm. They observed that upon overexpression of MSMEG_6752, *Msm* cells are not able to form pellicle biofilms. Furthermore, treatment of Mtb biofilms with cellulase MSMEG_6752 leads to disruption of the biofilms and release of glucose subunits (28168306). However, it remains to be determined whether cellulose is present in other models of Mtb biofilms, namely leukocyte-induced biofilms and pellicle biofilms. Additionally, the presence of cellulose should be analyzed in the biofilms of other mycobacterial species as besides Mtb and Msm. Another important type of biofilm that has not been studied in mycobacteria is the macrocolony. Cellulose has been shown to play a critical role in the architecture of the macrocolony morphology for *Escherichia coli* [122], but the role of cellulose in the macrocolony morphology of mycobacterium species remains to be established. Cellulose is currently detected in biofilms using dyes such as Congo red and Calcofluor-White. Although these methods are suitable, but more specific biologically encoded sensors of cellulose (such as a cellulose-binding domain coupled with a fluorescent tag) could help in the analysis of the architectural role of cellulose in biofilms. The demonstration of cellulose as a critical component of Mtb biofilms has opened new avenues of research. The genetic pathway used by Mtb for the synthesis of cellulose and their regulations are yet to be identified. Cellulose is synthesized in bacterial cells using a multiprotein complex called cellulose synthase with the core catalytic activity residing in the BcsA and BcsB proteins [123, 124]. Although a number of mycobacterial species, such as *M. neoaurum* (*Uniprot ID* - A0A024QK68), *M. cosmeticum* (Uniprot ID - W9B851), etc., contain genes encoding for putative components of the cellulose synthase, but the Mtb genome does not seem to encode either BcsA or BcsB. Interestingly, the Mtb genome encodes few cellulases [120], which could be expressed and secreted in a spatiotemporal manner to facilitate the regulated dispersal of biofilm residents. It is noteworthy that the Mtb genome encodes for a large number of glycosyltransferases [125], including many uncharacterized ones that may function as non-canonical cellulose synthase. Previously a number of approaches besides the sequence similarity-based method were utilized for identification of components of the cellulose synthase in other bacterial species. These include the use of activity-based enrichment and purification of the cellulose synthase complex [126] using the photo-affinity probe 5-azido-UDP-Glc [127], protein-protein interaction-based screening [128], and transposon insertion mutagenesis screens [129]. We believe that such approaches could lead to the identification of the cellulose synthase of Mtb as well. Identification of the genetic pathway/s involved in cellulose biosynthesis could further facilitate the analysis of the role of Mtb biofilms in TB pathogenesis. Interestingly, cellulose synthesis is regulated post-translationally via cyclic-di-GMP (c-di-GMP) [130]. C-di-GMP is synthesized by the diguanylate cyclase (dgc) enzymes with a characteristic GGDEF motif and is degraded by phosphodiesterase (pde) enzymes with an EAL or HD-GYP motif [131]. Mtb possesses both a dgc (Rv1354c) and pde (Rv1357c). Interestingly, Rv1354c has the GAF, GGDEF, and EAL domains organized in tandem and are able to synthesize and degrade c-di-GMP, whereas Rv1357c contains only the EAL domain and degrades c-di-GMP to pGpG [132]. Intriguingly, the pde deletion mutant of *M. bovis* BCG forms highly matured pellicle biofilms and colonies with higher levels of cording. In line with these findings, the pde deletion mutant survived better in immunocompetent mice [133]. On the contrary, a pde mutant of Mtb has decreased survival in THP-1 cells and in a mouse model [134]. Despite these exciting studies, the role of c-di-GMP in the regulation of cellulose synthesis and *in vivo* biofilm formation still needs to be explored. Importantly, TRS-induced biofilms seem to contain other polysaccharides, in addition to cellulose [108]. Further research is required for the isolation, purification, identification, and characterization of these polysaccharides. We believe that our understanding of mycobacterial biofilms will rapidly expand with the characterization of these polysaccharides. Additionally, the identification of the genetic pathways that contribute to polysaccharide synthesis and their regulation *in vitro* and *in vivo* represent exciting research opportunities.

### Proteins

A number of structural proteins play an important role in microbial biofilms. Structural proteins are known to facilitate the interaction of the bacterial cells with the substratum, the EPS of the biofilm, and with other bacterial cells. Mtb cells produce a number of adhesins such as fibronectin-binding proteins, heparin-binding hemagglutinin adhesin (HBHA), and pili. A few of these proteins have been shown to play important roles in biofilm formation or aggregation of Mtb cells. HBHA is a bacterial cell surface-associated protein that can also be secreted. Importantly, HBHA can induce auto-aggregation of Mtb cells at a concentration of 0.5 µg/ml [135]. Mtb pili encoded by Rv3312A are involved in the formation of pellicle biofilms of Mtb [136]. TRS-induced Mtb biofilms can be disintegrated by the use of proteases [108] suggesting that some yet-unidentified structural proteins play a critical role in maintaining biofilm integrity. Efforts towards the identification of such proteins should be made, as this holds the key to our capability to disrupt Mtb biofilms. The presence of HBHA or pili remains to be analyzed in TRS-induced Mtb biofilms. Interestingly, pili also play a critical role in the architecture of *E. coli* colonies [137]. The role of pili in the architecture and microanatomy of the Mtb colony remains to be analyzed. Besides containing structural proteins, EPS also has a number of enzymatic activities and quorumsensing molecules to enable communication among the resident cells. A number of reactive oxygen species (ROS)-detoxifying enzymes are secreted by biofilm resident cells. These enzymes protect the bacterial cells against ROS generators. However, the enzymes and quorum-sensing molecules of Mtb biofilms have not been characterized and represent an important research prospect.

### eDNA

Besides polysaccharides, eDNA is also known to be an important structural component of microbial biofilms [114]. As described earlier in this review, eDNA is found in the biofilms of *M. avium* [67], *M. abscessus*, and *M. chelonae* [69]. In context of Mtb biofilms, the presence of eDNA was reported in leukocyte lysate-induced and TRS-induced biofilms of Mtb [106, 108]. Degradation of eDNA using Dnase treatment led to the disruption of leukocyte lysate-induced Mtb biofilms but not of TRS-induced biofilms. These observations suggest different structural roles in the two models of Mtb biofilms. In TRS-induced biofilms, eDNA was observed at the stalk of the microcolonies. These observations suggest that cellulose, along with eDNA, could be involved in attaching the microcolonies to the substratum. In a number of biofilms, the eDNA originates through the altruistic self-killing of a few resident cells [138] or is actively released through membrane vesicles [139]. Currently, we do not know how the eDNA originates in Mtb biofilms and identification of these pathways will improve our understanding of the physiological functions of eDNA. It is important to mention that Mtb aggregates in response to interferon gamma [140]. Mtb is known to produce extracellular vesicles [141] and possesses 88 toxin-antitoxin systems [142], but their role in Mtb biofilms remains unknown. It will be interesting to analyze whether Mtb cells residing in biofilms utilize extracellular vesicles for secreting DNA. The study of the spatiotemporal expression of toxin-antitoxin modules would help us to understand their role in biofilm maintenance. In our view, taking advantage of the models of Mtb biofilms, further research should be focused on understanding the different steps of biofilm formation and maturation. It is plausible that, in response to yet-unidentified signaling molecules, Mtb cells start producing adhesins (or alternatively modulate the cell surface to increase surface hydrophobicity) that facilitate the attachment of bacterial cells to the substratum. After attachment of the Mtb cells to the substratum, more cells start adhering to the surface and start producing polysaccharides along with structural proteins. Other components such as DNA or enzymes are contributed through regulated lysis of a few cells or localized production of extracellular vesicles. The microcolony thus established grows bigger through recruitment of more cells or cell division of resident bacteria. Localized production of EPS-degrading enzymes such as cellulase, protease, and Dnase facilitate the dispersal of Mtb biofilms.

In summary, research on Mtb biofilms has gained momentum with the establishment of three different models of biofilm formation. Although cellulose has been characterized as a key constituent of Mtb biofilms, it is important that the nature of the EPS of Mtb biofilms is further characterized. The demonstration of the presence of Mtb biofilms in animals/humans will further advance research on Mtb biofilms; however, this will require identification of more specific biomarkers for Mtb biofilms. With the deeper understanding of the nature of Mtb biofilms, new interventions in therapy and diagnosis of TB can be facilitated.

## *IN VIVO* PERSPECTIVE OF MTB BIOFILMS

A hallmark of Mtb infection in humans is the presence of granulomas with a caseating necrotic core at the center. Interestingly, several extracellular Mtb cells were detected in the necrotic core and in the acellular rim of necrotic lesions [143]. These bacteria were present either as single cells or as cluster of bacteria encapsulated within a matrix of extracellular polymers. A fraction of bacteria residing in clusters at the necrotic core and acellular rim could survive treatment of guinea pigs with antimycobacterial drugs [143]. Given the critical observations it is tempting to speculate that Mtb may form biofilms in vivo. However, to conclusively demonstrate that these clusters of extracellular Mtb cells are indeed Mtb biofilms, the presence of bacteria-synthesized ECM surrounding these clusters is required. We have recently proposed that cellulose could be used as a marker for the detection of Mtb biofilms in infected animals and humans [108, 144]. Additionally, since humans do not produce cellulose, detection of cellulose in human or animal tissues surrounding mycobacterial cells could indicate the presence of Mtb biofilms *in vivo*. We believe that such a finding would be a major step forward in our understanding of mycobacterial physiology inside the host. It must be noted here that previous studies have also identified short-chain free mycolic acid variants as markers of Mtb pellicle biofilms; thus, the presence of these variants of mycolic acid in human or animal tissue around the Mtb cells could be suggestive of the existence of the Mtb biofilm *in vivo*. We presume that the demonstration of the presence of Mtb biofilms in animals or humans could be a milestone in the field.

## CONCLUDING REMARKS

A number of mycobacterial species such as Mtb and a few NTMs cause chronic infections, and their treatment requires the usage of multiple anti-mycobacterials for a long period of time. Such drug-tolerant chronic infections are often associated with *in vivo* biofilms. Emerging evidence suggests that few mycobacterial species make *in vivo* biofilms, thus understanding the bacterial physiology of mycobacteria residing in the biofilms and the nature of ECM is key to our ability to treat such infections. Recent studies have also suggested that free mycolic acids, glycopeptidolipids and other cell wall components could alter the cell-to cell interaction to influence biofilm formation. A few studies have also suggested a structural role of polysaccharides and extracellular DNA in maintaining the structural integrity of the mycobacterial biofilms. Importantly, cellulose has been proposed as the marker of Mtb biofilms. A few studies have also suggested that reductive stress (due to accumulation of reduced counterparts of a redox couple such as NADH or thiols) could initiate biofilm formation. In the view of these findings, further research exploring the genetic pathway used by mycobacterial species to form biofilms *in vitro* and *in vivo* and its regulation could be important contributions in the field.

## Abbreviations:

EPS: extracellular polymeric substance,
GPL: glycopeptidolipids,
HBHA: heparin-binding hemagglutinin adhesin,
MMDAG: monomeromycolyl diacylglycerol,
NTM: non-tuberculous mycobacteria,
TB: tuberculosis,
TDM: trehalose dimycolate,
TRS: thiol-reductive stress.
